# 237 ’Ireland Lights Up’—evaluating the reach of a community walking initiative across GAA clubs in Ireland: an implementation science study

**DOI:** 10.1093/eurpub/ckae114.137

**Published:** 2024-09-26

**Authors:** Nicola Briggs, Aisling McGrath, Niamh Murphy, Barry Lambe, Noel Richardson

**Affiliations:** South East Technological University, Waterford, Ireland; South East Technological University, Waterford, Ireland; South East Technological University, Waterford, Ireland; South East Technological University, Waterford, Ireland; South East Technological University, Waterford, Ireland

## Abstract

**Purpose:**

‘Ireland Lights Up’ (ILU) is a community-based walking programme which runs in over 1000 Gaelic Athletic Clubs (GAA) in Ireland every January. It consists of clubs turning on their floodlights on a designated evening to facilitate group walking events around the perimeter of the playing fields. Formative research has indicated that ILU can potentially increase physical activity (PA) and social connectedness. As part of a broader study, this research aims to capture the impact of ILU in diverse community settings across Ireland by measuring its current reach in participating clubs and identifying the most viable implementation strategies to improve potential reach to underserved and disadvantaged populations. This phase of the research will provide a comprehensive overview of the demographics and behaviours of both participants and non-participants in ILU, exploring the factors influencing reach.

**Methods:**

The broader study will employ a hybrid type-two effectiveness-implementation design utilising the RE-AIM and PRISM frameworks. This phase of the study will employ a mixed methodology, collecting qualitative data through observations in 10 - 12 implementing clubs and one-to-one semi-structured interviews with club leaders and participants. Quantitative data will be obtained through questionnaires distributed to all ILU-participating clubs, complemented by attendance records and manual club counts. Non-participating GAA clubs will also be interviewed to enhance understanding of reach.

**Results:**

The study will document the percentage of individuals in the community engaged in ILU, comparing participant characteristics to non-participants and the target population, particularly emphasising socially disadvantaged groups. The study will also report participant and stakeholder perceptions and expectations regarding reach.

**Conclusions:**

The overarching aim of the study is to develop a structured framework for involving marginalised, isolated, and vulnerable populations in physical activity within the context of sports club settings. To effectively implement strategies, a complete understanding of the demographics and behaviours of those involved is vital. The aim of this phase of the research is to discern and identify implementation strategies that advance both reach and participation.

**Funding Source:**

Funded by SETU PhD Scholarship Programme.

